# Case report: intracranial fungal granuloma in a dromedary (*Camelus dromedarius)*

**DOI:** 10.1186/s12917-026-05391-1

**Published:** 2026-03-06

**Authors:** Meike-Louisa Schmidt, Marlene Sickinger, Volker Rickerts, Ilka McCormick-Smith, Kernt Köhler

**Affiliations:** 1 Institute of Veterinary Pathology, Faculty of Veterinary Medicine, Justus- Liebig-University Giessen, Frankfurter Str. 96, 35392 Giessen, Germany; 2 Clinic for Ruminants and Herd Health Management, Faculty of Veterinary Medicine, Justus-Liebig-University Giessen, 35392 Giessen, Germany; 3 Konsiliarlabor für Kryptokokkose und seltene Systemmykosen, Robert Koch Institute, FG16, 13353 Berlin, Germany

**Keywords:** Aspergillosis, Aspergillus fumigatus, Dromedary, Fungal granuloma

## Abstract

**Background:**

Here, we describe a case of an intracranial fungal granuloma in an adult, non-castrated female dromedary camel (*Camelus dromedarius*).

**Case presentation:**

An 11-year-old female dromedary presented with progressive neurological signs over several weeks. Neurological examination suggested a forebrain lesion, and magnetic resonance imaging (MRI) revealed a mass in the frontal fossa. Owing to poor prognosis, the animal was euthanized. Histopathological examination demonstrated a granulomatous-necrotizing inflammatory process containing fungal structures. Broad-range polymerase chain reaction (PCR) and amplicon sequencing results were consistent with a member of the *Aspergillus fumigatus* species complex being the causative agent.

**Conclusions:**

Central nervous system involvement is rare in fungal infections. Intracranial fungal granulomas should be considered as a differential diagnosis in camels presenting with progressive neurological signs and space-occupying intracranial lesions.

## Background

Mold infections of the central nervous system (CNS) are rare in both human and veterinary medicine [1, 2]. When they occur, they are often associated with high morbidity and mortality, largely due to late diagnosis due to unspecific presentation and the limited penetration of antifungal agents across the blood-brain barrier [3]. *Aspergillus fumigatus* is a ubiquitous saprophytic mold fungus that acts as an opportunistic pathogen, posing a particular threat to immunocompromised individuals [4, 5]. In humans, aspergillosis most frequently affects patients undergoing chemotherapy or other conditions associated with immunodeficiency but also chronic lung conditions. More recently, invasive aspergillosis associated with viral pneumonia particularly in the context of severe influenza or COVID-19, has been recognized as a clinically significant complication [4].

In animals, *A. fumigatus* is a well-established pathogen in several species. Infections usually remain localized to the respiratory tract, causing sinusitis, pneumonia, or air sacculitis depending on the host species [5]. Dissemination to extra-respiratory sites is uncommon and usually occurs in immunocompromised animals or in certain predisposed breeds [1].

Diagnosis of aspergillosis in animals and humans can be challenging, as clinical signs are often nonspecific and fungal culture frequently yields negative results. Therefore, histomorphological demonstration of suggestive fungal elements in tissue, combined with molecular confirmation via broad-range quantitative PCR, is increasingly regarded as sufficient for establishing a diagnosis [6]. The term *Aspergillus fumigatus* species complex is an accepted designation that comprises several closely related Aspergillus species that cannot be reliably differentiated by morphology or routine PCR methods [7].

## Case presentation

An 11-year-old adult female dromedary (*Camelus dromedarius*), maintained as a circus animal, was presented for evaluation of progressive neurological signs of approximately three weeks duration. The animal exhibited compulsive hiking behavior and collided repeatedly with objects. Initial therapy by the attending veterinarian with antibiotics, analgesics, and vitamin supplementation was unsuccessful.

On clinical examination, the animal alternated between apathy and agitation, displaying compulsive walking, head pressing, and torticollis. Numerous abrasions were present across the body. Assessment of pupillary light reflexes was precluded because of the severe compulsive movements of the animal, however, droop and ciliary reflexes were intact. Hematological analysis revealed moderately elevated creatinine concentrations (243.5 µmol/L; reference range: 53–122 µmol/L); all other parameters were within normal limits.

The neurological examination findings suggested a forebrain lesion, indicating the need for advanced imaging. As magnetic resonance imaging (MRI) is considered the imaging modality of choice for the evaluation of intracranial disease, allowing accurate lesion localization and assessment of lesion extent, MRI of the head was performed under general inhalation anesthesia. A space-occupying mass within the frontal fossa causing marked mass effect on the adjacent forebrain was identified, with differential diagnoses including granuloma or neoplastic process.

Given the poor prognosis, euthanasia was elected and performed under general inhalation anesthesia by intravenous administration of pentobarbital sodium, and a postmortem examination was subsequently conducted. Postmortem cerebrospinal fluid analysis demonstrated elevated protein concentrations, with cytology revealing individual lymphocytes and lytic macrophages. Gross examination revealed multiple body abrasions, the absence of several teeth, multifocal chronic suppurative-necrotizing periodontitis, moderate hepatic lipidosis, multiple, irregularly shaped, black conglobates within the forestomach compartmental system containing visible fragments of cordage consistent with rope or hay net material, and a cystic lesion (1.5 × 1 × 1 cm) in one kidney.

**Fig. 1:**
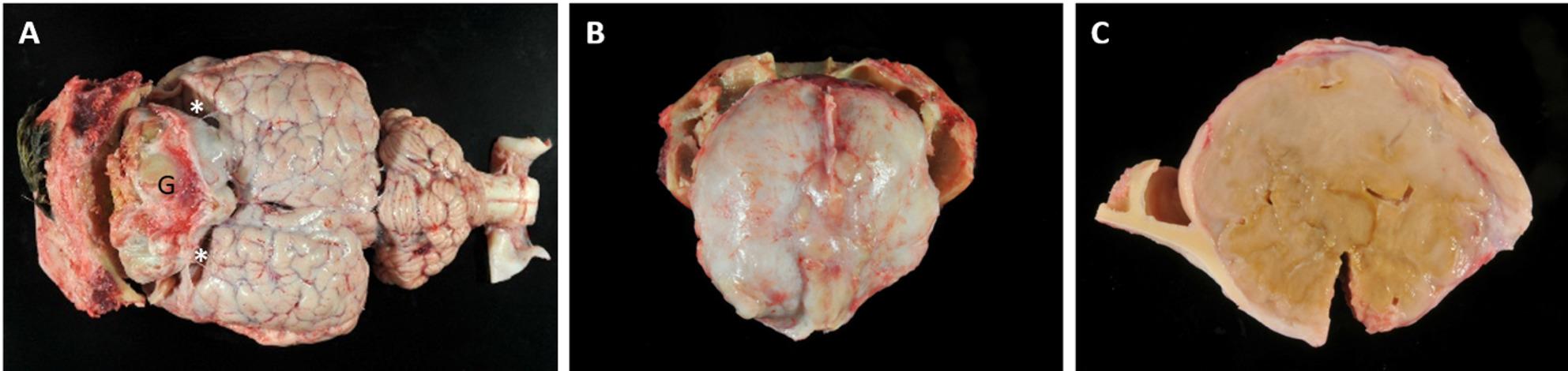
Macroscopical appearance of the mass. **A** 7 × 7 × 7 cm granuloma (G) causing compression atrophy of both frontal cerebral lobes (asterisks). **B** Extensive connection of the mass to the skull cap. **C** Coarse-elastic and partially mineralized tissue with a yellow-beige cut surface

Upon opening the skull, a well-circumscribed, beige, firm mass, measuring approx. 7 cm in diameter, was identified in the cranial calvaria, compressing both frontal cerebral lobes (Fig. 1). The mass was covered by leptomeninges and exhibited a coarse to mineralized texture on the cut surface.

**Fig. 2:**
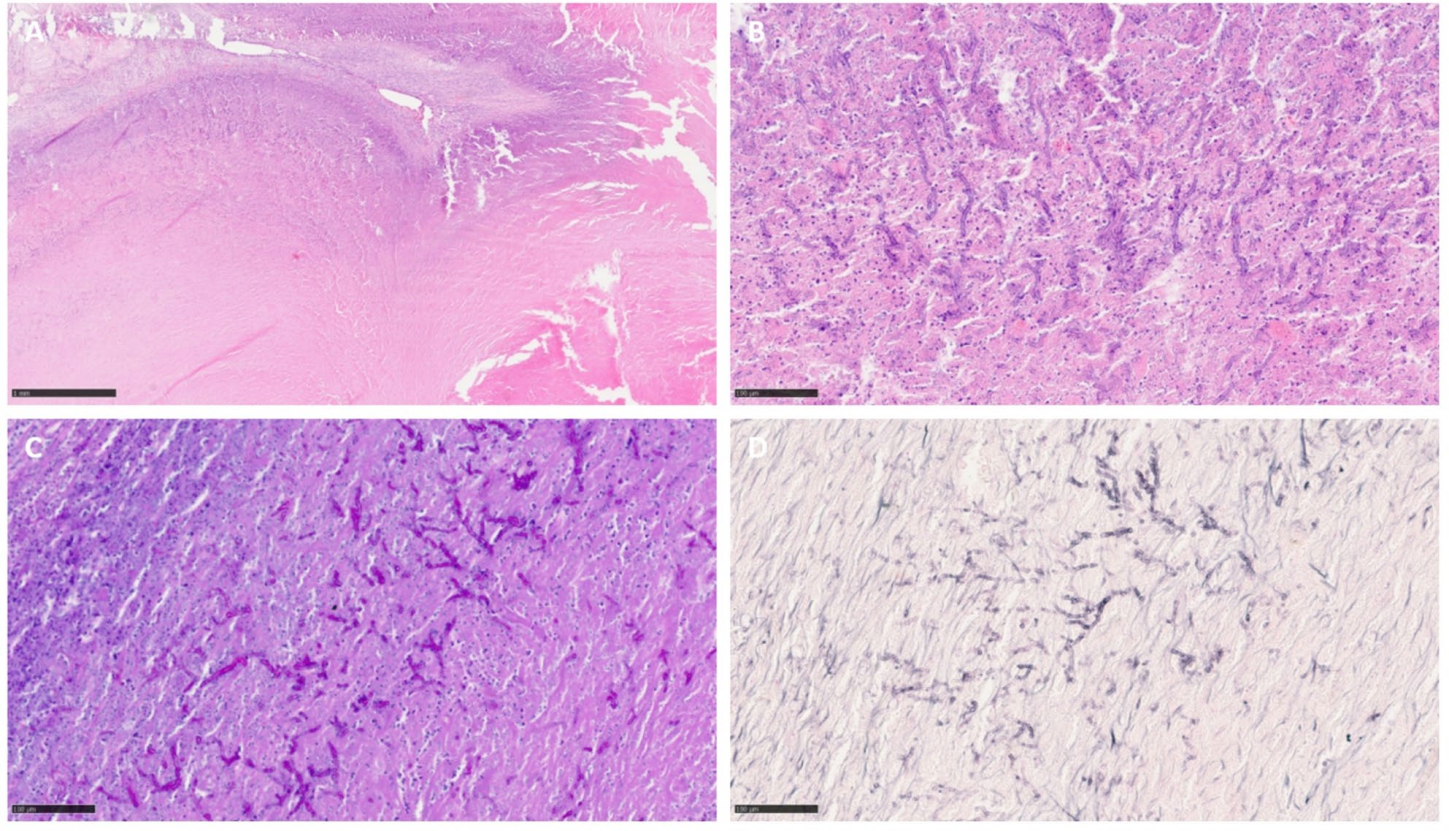
Histopathology of the mass. **A** Granuloma with areas of extensive coagulative necrosis and **B** numerous narrow, septate fungal hyphae with parallel walls, branching dichotomously at approximately 45° angles, morphologically suggestive of *Aspergillus* species (HE). **C** PAS reaction: positive. **D** Grocott stain: positive

Histopathologic examination revealed a granulomatous-necrotizing inflammatory process with abundant fungal hyphae (Fig. 2). The hyphae exhibited thin, parallel walls, dichotomous branching, and septation, and were positively stained with periodic acid-Schiff (PAS) reaction and Grocott stain (Fig. 2). Since the culture results were inconclusive, DNA was extracted from formalin-fixed, paraffin-embedded (FFPE) material and amplified via broad- range qPCR, which targeted the internal transcribed spacer (ITS) 2 region (Table 1) (8). The results of this assay, together with histomorphological findings, confirmed that the Aspergillus fumigatus species complex was the causative agent. 

**Table 1:** Primers used for broadrange qPCR

Primer name	Sequence (5’-3’)	Reference
5.8Sf	GTGAATCATCGARTCTTTGAAC	(9)
28S1r	TATGCTTAAGTTCAGCGGGTA	(9)

**Fig. 3:**
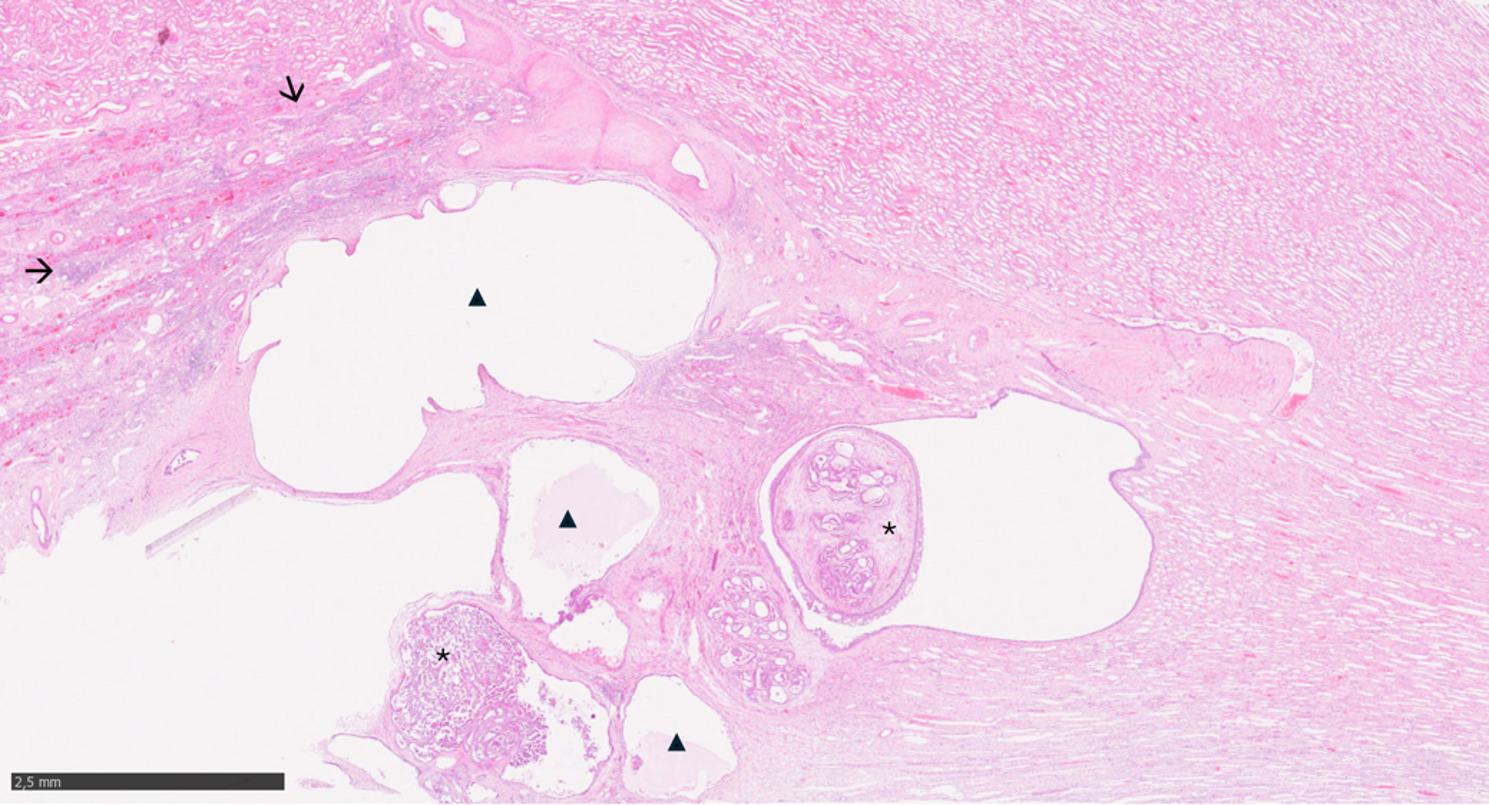
Histological section of the kidney. Areas of tubulo-papillary (asterisks) and cystic (arrowheads) carcinoma are present adjacent to regions of lymphohistiocytic interstital nephritis (arrows) (HE)

Additional histologic findings included mild gliosis within the frontal lobes and sparse mononuclear infiltrates within the leptomeninges. Other fungal lesions in other organs were not identified. As incidental finding, the renal cystic lesion was diagnosed as renal cell carcinoma (low grade), which displayed a partially tubule-papillary and partially cystic growth pattern (Fig. 3).

## Discussion

This report describes a case of a cerebral fungal granuloma caused by the *Aspergillus fumigatus* species complex in a dromedary camel. The diagnosis was supported by histopathological findings and further confirmed by broad-range qPCR and amplicon sequencing performed on FFPE tissue. Together, these complementary diagnostic approaches provided robust evidence supporting the final diagnosis [6].

The concurrent finding of renal cell carcinoma, while likely incidental, is noteworthy given its rarity in camelids [10]. The serum creatinine concentration was increased, which could be attributed to impaired renal function associated with the renal neoplasm.

*Aspergillus fumigatus* is a ubiquitous saprophytic mold and among the most frequently isolated species in clinical cases of aspergillosis in domestic animals. As an opportunistic pathogen, *A. fumigatus* can cause a broad spectrum of diseases, with severity and distribution influenced by the host’s immune status. In veterinary medicine, sinonasal infections are considered the most common form of aspergillosis. In dromedaries specifically, cases with multiorgan involvement [11] and one instance of scrotal aspergillosis [12] have been previously reported. Furthermore, there are descriptions of Aspergillus infections of the respiratory tract [13–15] as well as disseminated forms [16, 17] in New World camelids.

CNS involvement is exceedingly rare and can occur via several potential routes. In this case, direct extension from the nasal cavity or paranasal sinuses appears to be the most plausible pathway. Hematogenous spread is theoretically possible but seems unlikely here owing to the absence of lesions in other commonly affected organs such as the lungs or gastrointestinal tract. The animal was maintained as a circus animal. Its housing conditions and potential stressors are otherwise unknown. Concurrent systemic pathology may have contributed to immune dysregulation and increased susceptibility to opportunistic infection.

The animal presented with progressive, nonspecific neurological symptoms consistent with a forebrain lesion. While MRI provided anatomical localization and suggested the presence of a space-occupying mass, it could not distinguish between a granuloma or neoplastic process. Granulomas have been described in both human and veterinary medicine as lesions that can mimic various tumors on imaging, demonstrating that definitive differentiation based on imaging alone is often not possible [18]. In camels, other space-occupying intracranial masses reported include histiocytic sarcoma and meningioma [19]. Histopathology and special staining revealed characteristic septate, dichotomously branching hyphae, and molecular testing identified a member of the *Aspergillus fumigatus* species complex, underscoring the limitations of routine diagnostic techniques in species-level differentiation.

This report emphasizes the importance of including fungal granulomas in the differential diagnosis of intracranial mass lesions in camelids and other exotic species presenting with neurological signs. Treatment of CNS fungal diseases in large animals is rarely performed due to a lack of accredited drugs especially in food-producing animals, but increased awareness of such rare infections can support early recognition.

## Data Availability

All the data generated and/or analyzed during this study are available from the corresponding author upon reasonable request.

## Abbreviations

A.fumigatus: Aspergillus fumigatus
CNS: central nervous system
DNA: deoxyribonucleic acid
FFPE: formalin fixed paraffin embedded
ITS: internal transcribed spacer
MRI: magnetic resonance imaging
PAS: periodic acid-Schiff
PCR: polymerase chain reaction
qPCR: quantitative polymerase chain reaction
